# Development and Technical Validation of an Integrated Risk Calculator for Acute Coronary Syndrome Using ChatGPT-Assisted Coding

**DOI:** 10.7759/cureus.83410

**Published:** 2025-05-03

**Authors:** Musab Egaimi, Pierfrancesco Corvo, Hasan Al Houri, Ji Min Chang, Heeyoung Seo, Alghafek Almorraweh, Wonsuk Choi, Hassan Badreldin, Sahla Bashir, Mohamed H Serour, Lara Merghani, Mohammed Al Natour, Musab Mukhtar, Fabrizio Clementi

**Affiliations:** 1 Cardiovascular Center, Sheikh Khalifa Specialty Hospital, Ras Al Khaimah, ARE

**Keywords:** acute coronary syndrome, ai-assisted development, chatgpt coding, clinical decision support, grace score, integrated risk calculator, mehran score, risk stratification, technical validation, timi score

## Abstract

Introduction

Acute coronary syndrome (ACS) remains a major contributor to cardiovascular morbidity and mortality, necessitating efficient and accurate risk-stratification tools. Widely used models such as the Thrombolysis in Myocardial Infarction (TIMI), Global Registry of Acute Coronary Events (GRACE), and Mehran scores require separate manual inputs, leading to workflow inefficiencies and transcription errors. This study introduces an integrated digital tool that consolidates these scoring systems into a single interface and explores the feasibility of AI-assisted development in clinical software design.

Objective

The objective of this study is to develop and technically validate an integrated ACS risk calculator that streamlines the computation of TIMI, GRACE, and Mehran scores while demonstrating the utility of AI-assisted coding in clinical applications.

Methods

A web-based calculator was developed using Hypertext Markup Language (HTML)/JavaScript, with AI-assisted code prototyping via ChatGPT (o3-mini-high) (OpenAI, San Francisco, CA). The interface standardizes shared clinical variables for all three risk scores. Validation was conducted using 226 ACS cases from the Sheikh Khalifa Specialty Hospital registry. TIMI and GRACE scores generated by the tool were compared against Get With The Guidelines-Coronary Artery Disease (GWTG-CAD) registry values using Pearson correlation. The Mehran score was internally validated through manual review. Congestive heart failure was inferred using Killip class > I to align inputs across models.

Results

The tool showed complete agreement with registry-based TIMI and GRACE scores (TIMI: r = 1.000, p < 0.001; GRACE: r = 1.000, p < 0.001). Manually reviewed Mehran scores demonstrated consistent output. The calculator reduced data entry duplication and preserved computational accuracy.

Conclusion

This study validates an integrated ACS risk calculator that unifies three established models within a single digital tool. It enhances workflow efficiency and demonstrates the practical value of AI-assisted, clinician-led development in cardiovascular decision support. Further clinical and usability validation is warranted.

## Introduction

Acute coronary syndrome (ACS) remains a leading cause of cardiovascular morbidity and mortality worldwide, necessitating accurate risk stratification tools to guide clinical management and improve patient outcomes. Among the most widely used risk scores are the Thrombolysis in Myocardial Infarction (TIMI) score [1], the Global Registry of Acute Coronary Events (GRACE) score [2], and the Mehran score [3]. These models incorporate key clinical and laboratory parameters to predict short-term mortality, major adverse cardiac events (MACE), and procedural risks such as contrast-induced nephropathy (CIN).

Despite their clinical utility, a major limitation in practice is the need for separate data entry across multiple risk calculators, increasing the risk of transcription errors and workflow inefficiencies. To address this, we developed an integrated risk calculator that consolidates the TIMI, GRACE, and Mehran scoring systems into a single interface, aiming to streamline risk assessment and improve clinical workflow efficiency.

This study leverages data from the Get With The Guidelines-Coronary Artery Disease (GWTG-CAD) registry, an initiative by the American Heart Association (AHA) designed to enhance ACS outcomes and adherence to guideline-directed therapy [4]. The GWTG-CAD registry provides a structured national framework for quality improvement in cardiovascular care, systematically capturing clinical data, including TIMI and GRACE scores. While our integrated risk calculator incorporates TIMI, GRACE, and Mehran scores, validation of TIMI and GRACE was performed using the GWTG-CAD dataset, whereas Mehran score validation was explored through selective manual extraction and comparison of random patient samples.

Additionally, this study highlights the feasibility of clinician-led software development, utilizing AI-assisted coding tools such as ChatGPT (o3-mini-high) (OpenAI, San Francisco, CA) to facilitate rapid prototyping and deployment [5]. By integrating multiple risk stratification models into a single interface, we aim to improve accuracy, efficiency, and accessibility in ACS risk assessment.

## Materials and methods

Study design

We conducted a technical validation study comparing the integrated tool’s performance against conventional TIMI and GRACE calculations. The calculator captures all three scores (TIMI, GRACE, and Mehran); however, validation was conducted for TIMI and GRACE using the GWTG-CAD dataset. The Mehran score is not captured in GWTG-CAD and was therefore validated separately against values retrieved from the hospital information system (HIS).

Tool development

**Figure 1:**
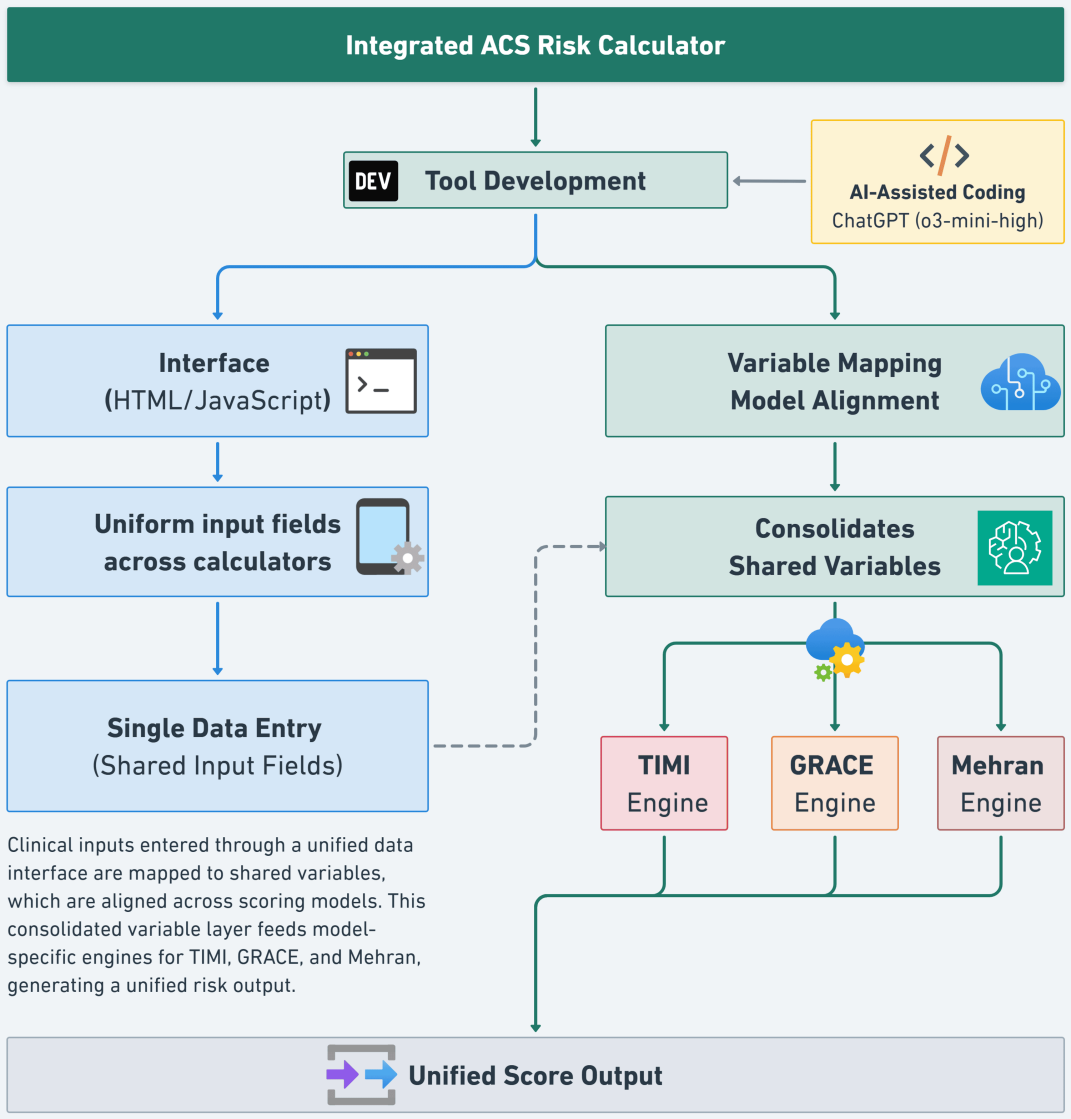
Figure created by the authors to illustrate the workflow schematic of the Integrated ACS Risk Calculator. The tool was developed using AI-assisted coding (ChatGPT o3-mini-high) and combines a standardized interface, built with HTML/JavaScript, with a unified backend logic for real-time calculation of TIMI, GRACE, and Mehran scores. Shared clinical variables are entered once and mapped across risk models via a consolidated data layer, enabling efficient and consistent risk stratification. ACS - Acute Coronary Syndrome; GRACE - Global Registry of Acute Coronary Events; HTML - Hypertext Markup Language; TIMI - Thrombolysis in Myocardial Infarction

A web-based Hypertext Markup Language (HTML)/JavaScript interface was designed to unify TIMI, GRACE, and Mehran scores into a single workflow. Overlapping clinical parameters were mapped into a streamlined data entry process, reducing redundancy and minimizing errors. The tool follows a structured input-to-output mapping system, allowing simultaneous calculation of multiple risk scores with minimal user input. Overlapping variables across TIMI, GRACE, and Mehran are entered once, optimizing efficiency and ensuring computational accuracy. The decision logic automates data processing, minimizes manual errors, and enables real-time risk assessment (Figure 1).

To ensure compatibility across scoring systems, we implemented standardized variable mappings where clinically appropriate. For the Mehran score, congestive heart failure (CHF) was inferred based on Killip class, with CHF points assigned for Killip class > I (i.e., II-IV). This approach enabled harmonized data capture while maintaining alignment with the original Mehran definition of CHF as New York Heart Association (NYHA) class III/IV or pulmonary edema. Given the established use of Killip classification in ACS risk models and its ability to capture bedside evidence of pulmonary congestion, it serves as a pragmatic, if not direct, substitute in the acute setting where functional NYHA assessment is infeasible.

**Figure 2:**
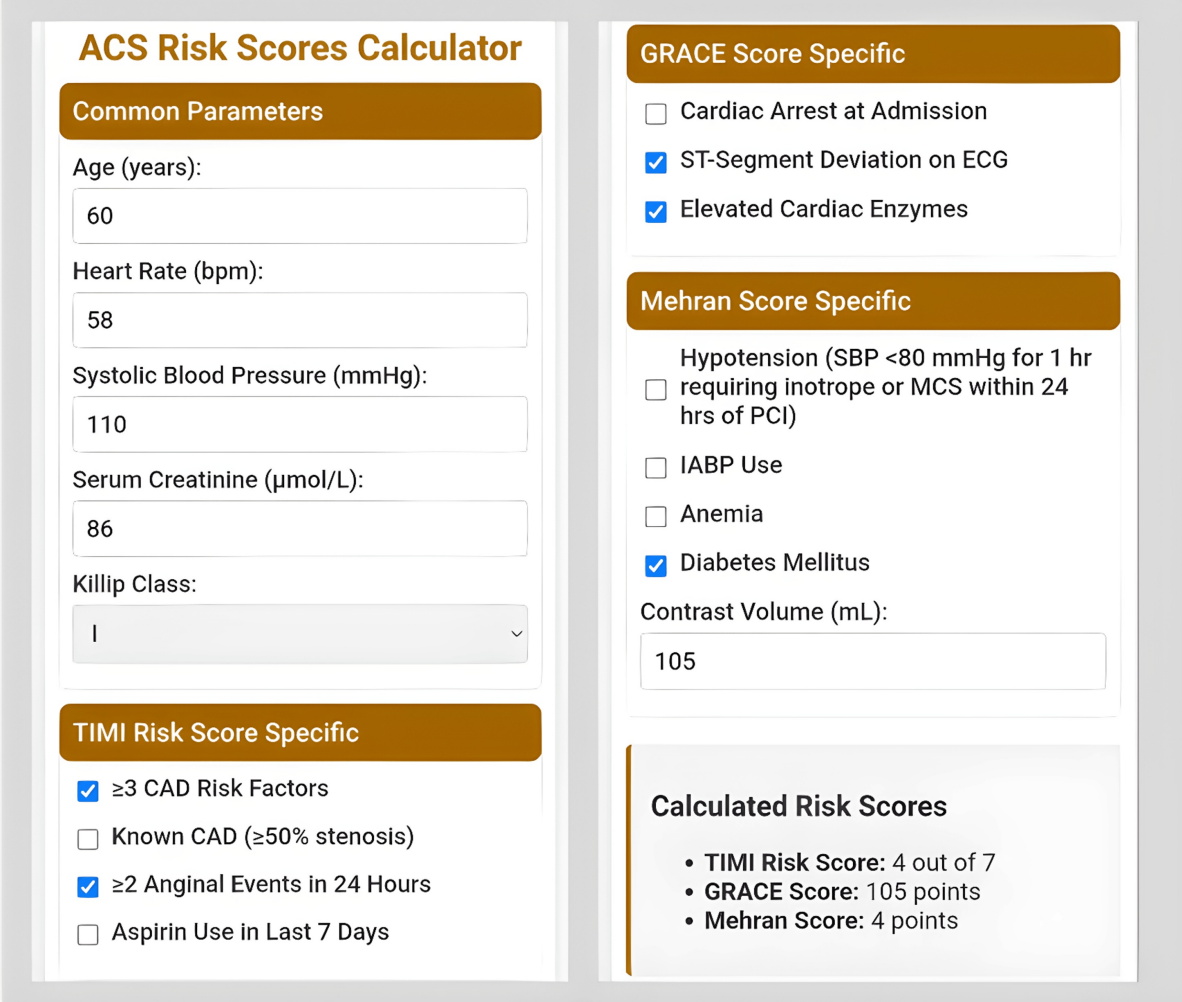
User interface of the Integrated ACS Risk Calculator developed in this study. The screenshot illustrates a streamlined, single-entry system for calculating TIMI, GRACE, and Mehran scores. The design minimizes redundant data entry and consolidates outputs into a unified interface, improving clinical workflow. ACS - Acute Coronary Syndrome; CAD - Coronary Artery Disease; GRACE - Global Registry of Acute Coronary Events; TIMI - Thrombolysis in Myocardial Infarction

ChatGPT (o3-mini-high) was leveraged to assist in coding, debugging, and refining the tool’s user interface, demonstrating the potential of AI-driven clinician-led innovation [6]. The integrated system provides a single-point data entry, eliminating the need for repeated manual input and improving workflow efficiency (Figure 2).

Technical validation

A de-identified dataset of 226 ACS patients admitted to Sheikh Khalifa Specialty Hospital (SKSH) between 2022 and 2023 was used for validation. For each patient, we computed TIMI and GRACE using the integrated tool and compared them against the values extracted from the GWTG-CAD registry methods. A Pearson correlation analysis was performed to assess the consistency between captured values and recalculated values using the integrated tool. 

Because the Mehran score is not captured in our GWTG-CAD dataset, validation was performed on a subsample of 30 patients - selected to span the observed score distribution - whose required clinical variables (age, baseline creatinine, contrast volume, Killip class, etc.) were extracted from the HIS and input into the calculator, then cross-referenced against the Mehran score already calculated and stored in the HIS. A working version of the calculator, including all source HTML/JavaScript, is publicly accessible (see Appendices).

## Results

**Figure 3:**
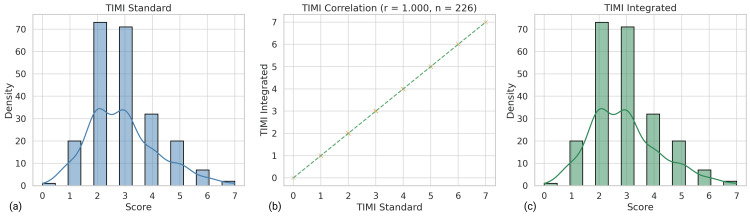
Visual validation of TIMI score agreement between the standard method and the integrated ACS Risk Calculator. (a) Distribution of TIMI scores from the standard method based on registry values. (b) Scatter plot illustrating perfect correlation (r = 1.000, n = 226) between integrated and standard TIMI scores. (c) Distribution of TIMI scores generated by the integrated calculator. These panels confirm that the integrated tool reproduces TIMI scores with full fidelity across the dataset. ACS - Acute Coronary Syndrome; TIMI - Thrombolysis in Myocardial Infarction; r - Pearson correlation coefficient; TIMI Standard - TIMI score calculated using the standard method; TIMI Integrated - TIMI score generated by the integrated calculator

**Figure 4:**
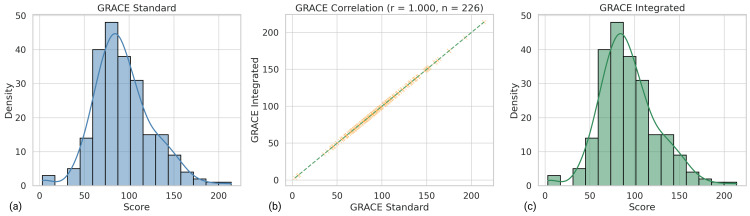
Visual validation of GRACE score agreement between the standard method and the integrated ACS Risk Calculator. (a) Distribution of GRACE scores calculated using the standard method from the registry dataset. (b) Scatter plot showing perfect correlation (r = 1.000, n = 226) between GRACE scores computed by the integrated tool and the registry-based standard values. (c) Distribution of GRACE scores generated by the integrated tool. All subpanels demonstrate full computational agreement, confirming the integrated tool's accuracy. ACS - Acute Coronary Syndrome; GRACE - Global Registry of Acute Coronary Events; GRACE Standard - GRACE score calculated using the standard method; GRACE Integrated - GRACE score generated by the integrated calculator; r - Pearson correlation coefficient

Among the 226 patient records analyzed, every TIMI and GRACE value produced by the calculator matched its corresponding registry entry exactly. This identity is reflected in the Pearson coefficients (TIMI r = 1.000, p < 0.001; GRACE r = 1.000, p < 0.001), as shown in Figure 3 and Figure 4, confirming that the tool reproduces the arithmetic logic of both scores. A random, blinded audit of 30 cases for the Mehran algorithm likewise revealed 100% concordance between manual and automated calculations, supporting accurate implementation across all three models.

While perfect correlation demonstrates numeric equivalence, it does not by itself exclude subtle systematic bias. Accordingly, a follow-up phase will apply full agreement analyses, including intraclass-correlation coefficients and Bland-Altman plots, to complement these initial findings and provide a more granular assessment of measurement fidelity.

## Discussion

This study demonstrates that an integrated risk calculator can successfully consolidate TIMI, GRACE, and Mehran scores into a single tool, reducing redundant data entry while maintaining the accuracy of validated risk models. By eliminating multiple data entry points, the tool enhances workflow efficiency, mitigates transcription errors, and facilitates faster clinical decision-making.

Technical validation demonstrated exact replication of TIMI and GRACE scores (Pearson r = 1.000 for both; p < 0.001) and 100 % concordance in a blinded audit of 30 Mehran cases, confirming the calculator’s computational accuracy across all three risk models. However, perfect correlation alone does not preclude subtle systematic bias, highlighting the importance of subsequent agreement studies, such as intraclass‐correlation coefficients and Bland-Altman analyses, to deepen assessment of measurement fidelity. Future work will focus on evaluating real-world clinical adoption, including time savings and user experience studies.

To pragmatically implement the heart failure component of the Mehran score in a real-time digital tool, we used Killip class > I as a surrogate for CHF. Although the original Mehran model defines CHF as NYHA class III/IV or pulmonary edema, NYHA classification is impractical at initial presentation, whereas Killip class is routinely applied and reflects bedside assessment of pulmonary congestion and hemodynamic compromise. This substitution is supported by studies showing that Killip class ≥2 correlates with objective markers of acute heart failure and predicts post-discharge CHF risk. For example, in the INFUSE-AMI trial, Killip class ≥2 at presentation was an independent predictor of NYHA class ≥2 symptoms at 30 days, which were in turn associated with a four-fold increase in heart failure hospitalization and mortality at one year [7]. Similarly, in the AMI-Kyoto Risk Study, Killip class ≥2 patients undergoing percutaneous coronary intervention (PCI) exhibited significantly higher in-hospital mortality and rates of left ventricular dysfunction, hallmarks of NYHA class III/IV CHF [8]. Additionally, Sgura et al. demonstrated that higher Killip class and CHF presence at admission were strong predictors of long-term rehospitalization for heart failure and adverse outcomes following primary PCI, directly validating the CHF component of the Mehran score in this clinical context [9].

Moreover, this study highlights the growing role of AI-driven software development in clinical decision support. The successful utilization of ChatGPT (o3-mini-High) in the development process enabled rapid code generation, real-time debugging, and refinement of the tool. These findings align with recent studies emphasizing the potential of AI to accelerate clinician-led innovation in medical informatics [10].

While the integrated tool improves efficiency, future research should evaluate its clinical impact on decision-making and patient outcomes. Additionally, efforts should be made to integrate the calculator into electronic health records (EHRs) to further streamline risk assessment in hospital settings.

Limitations

This study focused exclusively on technical validation, assessing the integrated tool’s ability to replicate existing scores; it did not evaluate clinical outcomes, decision-making impact, or usability in real-time practice. Validation of Mehran scores was limited to a small subset of manually extracted records and was not conducted at scale; comprehensive validation remains a future direction. Additionally, while Killip class was used as a practical proxy for NYHA III/IV CHF, further validation comparing Killip and NYHA equivalence in this context would strengthen this mapping.

## Conclusions

We successfully developed and technically validated an integrated ACS risk calculator that consolidates the TIMI, GRACE, and Mehran scoring systems into a single digital platform. The tool demonstrated full statistical consistency with conventional methods for TIMI and GRACE scores, and manual review confirmed accurate implementation of the Mehran scoring logic. By eliminating redundant data entry and harmonizing overlapping clinical variables, the tool improves workflow efficiency and supports real-time risk assessment in ACS settings. The integration of the Killip class as a surrogate for heart failure enables standardized input across models while preserving clinical coherence.

This study also highlights the feasibility of clinician-led, AI-assisted development in the design of decision-support tools. Future work should focus on clinical validation, real-world adoption, and integration with EHR systems to maximize the tool’s impact in practice.

## Appendices

A working version of the integrated ACS risk calculator is publicly accessible. It can be accessed at https://egaimi.github.io/acscalc/.
